# Municipal return to work management in cancer survivors undergoing cancer treatment: a protocol on a controlled intervention study

**DOI:** 10.1186/s12889-015-2062-1

**Published:** 2015-07-29

**Authors:** Christina M. Stapelfeldt, Merete Labriola, Anders Bonde Jensen, Niels Trolle Andersen, Anne-Mette H. Momsen, Claus Vinther Nielsen

**Affiliations:** Public Health and Quality Improvement - CFK, Central Denmark Region, MarselisborgCentret, P.P. Oerums Gade 11, Building 1B, 8000 Aarhus C, Denmark; Department of Oncology, Aarhus University Hospital, Aarhus, Denmark; Section of Biostatistics, Department of Public Health, Aarhus University, Aarhus, Denmark; Section of Clinical Social Medicine and Rehabilitation, Department of Public Health, Aarhus University, Aarhus, Denmark

**Keywords:** Acceptance and commitment therapy, Cancer survivor, Controlled trial, Individual placement and support, Intervention, Occupational rehabilitation, Readiness for return to work, Social inequality, Workplace

## Abstract

**Background:**

Cancer survivors are often left on their own to deal with the challenges of resuming work during or after cancer treatment, mainly due to unclear agreements between stakeholders responsible for occupational rehabilitation. Social inequality exists in cancer risk, survival probability and continues with regard to the chance of being able to return to work.

The **aim** is to apply an early, individually tailored occupational rehabilitation intervention to cancer survivors in two municipalities parallel with cancer treatment focusing on enhancing readiness for return to work.

**Methods/Design:**

In a controlled trial municipal job consultants use acceptance and commitment therapy dialogue and individual-placement-and-support-inspired tools with cancer survivors to engage them in behaviour changes toward readiness for return to work. The workplace is involved in the return to work process.

Patients referred to surgery, radiotherapy or chemotherapy at the Oncology Department, Aarhus University Hospital, Denmark for the diagnoses; breast, colon-rectal, head and neck, thyroid gland, testicular, ovarian or cervix cancer are eligible for the study. Patients must be residents in the municipalities of Silkeborg or Randers, 18–60 years of age and have a permanent or temporary employment (with at least 6 months left of their contract) at inclusion. Patients, for whom the treating physician considers occupational rehabilitation to be unethical, or who are not reading or talking Danish are excluded. The control group has identical inclusion and exclusion criteria except for municipality of residence.

Return to work is the primary outcome and is indentified in a social transfer payment register. Effect is assessed as relative cumulative incidences within 52 weeks and will be analysed in generalised linear regression models using the pseudo values method. As a secondary outcome; co-morbidity and socio-economic status is analysed as effect modifiers of the intervention effect on return to work.

**Discussion:**

The innovative element of this intervention is the timing of the occupational rehabilitation which is much earlier initiated than usual and the active involvement of the workplace. We anticipate that vulnerable cancer survivors will benefit from this approach and reduce the effects of social inequality on workability.

**Trial registration:**

Current Controlled Trials ISRCTN50753764. Registered August 21^st^, 2014.

## Background

### Cancer and return to work

Cancer prevalence has increased worldwide in recent years due to improvements in diagnostic procedures and treatment [1]. Approximately 40 % of new cancers are found in the working population [2], of whom many are motivated for and expect to return to work (RTW) [3], as RTW constitutes “normalcy”, has impact on quality of life and economic independence [4, 5]. However, the cancer disease and the treatment still increases the risk of early withdrawal from the labour market [6, 7]. Even though work is perceived as an important factor in quality of life [8, 9], for some cancer survivors work participation may be deprioritised by other ways of active participation in society [10]. Therefore knowledge on how cancer survivors value their work and how this affects the RTW process is needed. Among cancer survivors who do RTW some may face both health-related and work-related difficulties leading to reduced workability and recurrence of sick leave [11], which further increases the risk of prematurely exit from the labour market [12]. Some consistency exists in the literature about which factors are associated with RTW despite adverse side-effects from cancer treatment [13]. However, often cancer patients are left on their own to deal with these challenges [11, 14, 15, 10]; partly due to unclear agreements between stakeholders responsible for occupational rehabilitation [10, 16] and partly due to reluctance and fear of articulating work and RTW to a cancer survivor [17].

Research on RTW in cancer survivors has in recent years increased, partly because survival rates have increased [18, 19], and because patients with advanced, incurable disease are able to perform some kind of work, and will often prefer to stay on the labour marked as long as possible. Nevertheless, there still is a need for rigorous studies applying a randomised controlled trial design, with thorough information on intervention components and data reporting [19]. Due to the inconsistent conclusions drawn from the existing RTW studies in cancer survivors; evidence from research conducted on musculoskeletal and/or mental-related sickness absentees can give insights into effective RTW interventions in general and guide researchers in putting hypotheses forward on which elements cancer-oriented RTW interventions may contain. Thus, the provision of occupational rehabilitation to cancer survivors that consists of a combination of general and cancer specific intervention elements may prove to be an effective approach.

Some evidence supports that RTW interventions targeted musculoskeletal-related [20] or depressed absentees [21] are more effective when they address the workplace than interventions not targeting the workplace. Thus, in the planning of the present study it was crucial to incorporate involvement of the workplace as one of the key elements in the intervention. In a cancer setting it has been shown that employer and colleague support as well as work accommodation increases the RTW rate and reduces the likelihood of cancer-related work impairments after RTW [13].

Problem-solving-therapy interventions towards employees sick-listed due to adjustment disorders enhanced partial RTW compared to a non-guideline based care [22]. Cancer survivors may also display adjustment disorders due to difficulties in coping with having a cancer diagnosis along with expectations from relatives and friends, workplace and health care professionals in the RTW process. Fear of cancer recurrence may lead to fear avoidance behaviour, which limits participation in life - including work [23, 24]. Therefore, the identification of cancer survivor-experienced barriers regarding RTW and assistance in barrier modification were important elements that the present intervention should include.

Feuerstein et al. developed the Work and Cancer Model based on an extensive review of the literature [25]. A variety of factors coexists and may act both as facilitators and inhibitors of RTW among cancer survivors; individual characteristics, health and well-being, symptoms, function, work demands, work environment and finally structural factors [25]. There seems to be agreement in the literature that RTW-interventions in general should target the multi-dimensional factors that are associated with sickness absence and that seems also to be true in a cancer specific context. This perspective is in part substantiated in the Cochrane review by de Boer et al. on RTW interventions to cancer survivors. Although the studies had low quality it was concluded that one-dimensional interventions, i.e. psychological, physical or medical interventions did not improve RTW compared to care as usual, whereas a moderate quality evidence was found for a multidisciplinary approach (involving physical, psychological and vocational elements) compared to care as usual [18].

Cancer survivors in low socioeconomic groups may in particular experience the RTW-process difficult and tend to be at risk of recurrent sickness absence, unemployment or permanent withdrawal from the labour market [7, 26, 27]. It is not clear which role socioeconomic status plays on cancer survivors’ RTW-process, but unfavourable work conditions characteristic for low income and low-level educational jobs may explain some of this inequality [28]. Therefore work accommodations and supervisor support may be of particular importance in occupational rehabilitation in cancer survivors [3, 10, 29] to help cancer survivors overcome the imbalance between health and work demands.

Despite improved long-term survival rates for cancer patients, survivorship does not mean living without health complaints, but rather living with a chronic disease [30]. Cancer survivors do have an increased risk of recurrent cancers along with co-morbidities like cardiovascular disease, diabetes, osteoporoses etc. [31]. This further increases the risk for reductions in workability and calls for collaboration between the occupational rehabilitation stakeholders to enhance the chance of sustainable RTW.

### The Danish healthcare system

Permanent residents in Denmark pay approximately 40-50 % taxes of their income, by which almost all examinations and treatments within the Danish healthcare system are free of charge. The Danish Health and Medicines Authority is responsible for the organizational and clinical standards for the diagnostics and treatment for all cancer types, i.e. integrated cancer pathways [16]. The objective of the pathways is to reduce referral time, obtain faster diagnosing and early onset of treatment. To accomplish these general goals the pathways are operationalised in several Disease Management Programmes for Cancer of which one defines Rehabilitation and Palliation in Cancer [16]. The specific aim of cancer rehabilitation is, besides optimizing the patient's physical, psychological and social functioning while countering the limitations imposed by the side effects of cancer treatments and/or co-morbid conditions, also to offer occupational and vocational rehabilitation [32]. Few studies have been conducted on occupational rehabilitation offered parallel to cancer treatment at the hospital [33, 34], however more studies are ongoing/in preparation [35–37]. Thus, knowledge is scarce on whether occupational rehabilitation applied early and parallel to cancer treatment facilitates the RTW-process for cancer survivors. To our knowledge, no studies have been conducted in a Danish setting or in other Nordic countries, which has similar tax-financed health care systems as Denmark.

### Sickness absence management in Denmark

According to the Danish Sickness Benefit Act [38], the municipal job centres are responsible for paying sickness benefits and initiating occupational/vocational rehabilitation to help sick-listed persons to RTW.

All employed, self-employed, temporarily employed and unemployed persons fulfilling the criteria of previous employment (minimum 74 h within a period of 8 weeks) are eligible for sickness benefits.

According to law, the employer pays sickness benefits during the first 4 weeks, afterwards the municipality refunds the employer’s wage expenses for a maximum period.

The regulations for sickness benefits have been subject to several changes [39] and continue to be so. When the study started the maximum period was 52 weeks within a period of 78 weeks. From July 2014 the maximum period in general was reduced to 26 weeks. However, extensions can be granted and for persons suffering from cancer the maximum period may be unlimited.

Medical certificates were not mandatory but could be requested by the municipal social security system and the employer. However, from January 2015 a medical certificate became obligatory after 8 weeks of sickness absence.

After the newest reform in 2014–15 the sickness beneficiaries are assigned into three categories: Category 1 includes persons who are likely to RTW within eight weeks without intervention. Category 2 includes persons who are unlikely to RTW within eight weeks unless activities facilitating RTW are initiated, i.e. coping-sessions and graded RTW. Category 3 includes persons who are unlikely to RTW within eight weeks unless multidisciplinary rehabilitation is implemented. This is planned by a municipal rehabilitation team within 12 weeks.

In accordance with the law sickness benefit officers are obliged to conduct regular follow-up interviews at least every four weeks with beneficiaries in category 2 and 3. Thus, municipal officers have been appointed the role as case managers, whereas employers have little responsibility for sickness absence management after they have had the first obligatory meeting with the employee after four weeks of sick-listing.

Most cancer survivors may be assigned in category 2, but in reality they are often spared the obligatory meetings with the social security officer and activities initiated by the job centre while receiving their treatment. This leads to a short time frame in which the cancer survivors are offered vocational/occupational rehabilitation; i.e. time between the end of treatment and the maximum period of sickness benefit reimbursement. Offering early occupational rehabilitation parallel to cancer treatment focusing on preparations for RTW and making arrangements together with the employer regarding work accommodations, graded RTW etc. should improve the RTW-process and reduce the recurrent sickness absence after RTW.

### Readiness for return to work scale

RTW after long-term sickness absence may be seen as a behavioural change or a process in several stages [40], depending on factors of which the person’s own RTW perceptions are predictive of future work participation [41–43]. The Readiness for RTW (R-RTW) scale [40] is based on the original stages of change model [44], which have been applied to various behaviours and across diverse disorders [45].

The R-RTW model addresses the motivational and social factors contributing to RTW behaviour and maintenance of work participation [46]. According to this model, the person progresses through stages of behaviour change i.e. RTW after sickness absence, shifting from the intention not to engage in RTW behaviour in the foreseeable future to a stage with initiating behaviour change, to maintain behaviour change and to RTW in a sustainable fashion.

Based on the score of the R-RTW scale it is possible to identify a person’s stage of readiness for change with regard to RTW allowing professionals, e.g. job consultants to tailor effective and individual support.

### Aim and hypotheses

The objectives are to apply an early, individually tailored occupational rehabilitation intervention to cancer survivors in two municipalities parallel with cancer treatment focusing on enhancing the R-RTW.

Two hypothesises;

- this early intervention conducted by a municipal job consultant will enhance the readiness for RTW and thereby increase the chance of RTW among cancer survivors from two municipalities compared with cancer survivors from municipalities not receiving this intervention but treated at the same hospital and receiving the standard municipal sick leave management.
- that socioeconomic status and/or co-morbidity will modify the effect of the intervention on RTW.

## Methods/Design

### Trial design

The study is a controlled trial conducted on cancer survivors treated at the Oncology Department, Aarhus University Hospital, Denmark with a 12 months follow-up.

### Participants

#### Intervention group

Cancer patients diagnosed with breast, colon-rectal, head and neck, thyroid gland, testicular, ovarian or cervix cancer referred to surgery, radiotherapy or chemotherapy at the Oncology Department at Aarhus University Hospital. Patients must be residents in the municipalities of Silkeborg or Randers, be at the age of 18–60 years and have a permanent or temporary employment (with at least 6 months left of their contract) at the inclusion date. Patients are eligible for participation whether they are still working or being on sick leave.

Patients, for whom the treating physician considers occupational rehabilitation to be unethical, or who are not reading or talking Danish are excluded.

Recruitment started in December 2013 and continues until the required sample size is obtained.

#### Control group

The control group is defined by patients with identical inclusion and exclusion criteria as the intervention group except that they live in other municipalities than Silkeborg and Randers.

The control group is identified via the electronic patient records at the Aarhus University Hospital.

### Recruitment of the intervention group participants

Patients are recruited to the intervention group in a three-stage process:

- At each cancer ward a nurse or assistant nurse assigned to this project identifies patients living in Randers or Silkeborg municipality with the eligible cancer diagnosis.
- The treating oncologist is notified of this patient and at first given consultation the patient is informed about the project, is handed a written information sheet also containing an informed written consent formula. The patient is asked to read the information sheet and is informed that a municipal job consultant will contact the patient at home by phone.
- The municipal job consultant may also identify eligible patients even before cancer treatment has begun because of sick leave spells exceeding 8 weeks. In that case the treating hospital ward is notified about inclusion status; in that way no cancer survivor is asked twice about inclusion.
- Finally the patient is telephoned by the municipal job consultant, who thoroughly informs the patient about the project. Participation includes the patients’ willingness to involve next of kin, employer, and colleagues. If the patient accepts participation the first meeting is set up and the informed written consent formula is signed on that occasion.

### Intervention

#### Control group

Cancer survivors in the control group will receive the usual municipal sickness benefit management within their municipal of residence as described in the background.

#### Intervention group

In both intervention municipalities the two job consultants attended a 4-days course in which they were specially trained by a psychologist in using elements of the Acceptance and Commitment Therapy (ACT) [23, 47] and the Individual Placement and Support Model (IPS) [48, 49].

During the inclusion- and intervention period the job consultants receive supervision by this psychologist once every month to secure a high degree of compliance with the intervention protocol.

#### Acceptance commitment and therapy

ACT is a development of cognitive behaviour therapy, which is based on recognition of the person’s values and immediate needs in current life situation. The goal of ACT is to increase psychological flexibility, which can be defined as the ability to contact the present moment more fully as a conscious human being, and to change or persist in behaviour that support one’s own values. Psychological flexibility is established through six core ACT processes: acceptance, cognitive fusion, being present, self as context, values, and committed action. Each of these areas is conceptualized as a positive psychological skill. The survivors are through dialogue with the job consultant confronted with these six core elements in order to enhance commitment and change in behaviour towards RTW [23].

#### Individual placement and support

The IPS inspired the way the job consultants systematize the actions by which they support RTW. The model originates from helping people with severe psychiatric illness to engage in paid employment, and builds on the following key elements: integration at the workplace, paid work, individualized services, and an ongoing individualised support [48, 49]. In this intervention the individualised support is “operationalised” in a number of phases, which correspond to the R-RTW obtained stages of change, i.e. a set of IPS-inspired actions are initiated according to the defined stage in which the survivor perceives him-/herself to be in: Pre-contemplation, Contemplation, Preparation for action - self-evaluative, Prepared for action – behavioural, Uncertain maintenance, or Proactive maintenance.

### Assessment of occupational rehabilitation needs

**Fig. 1:**
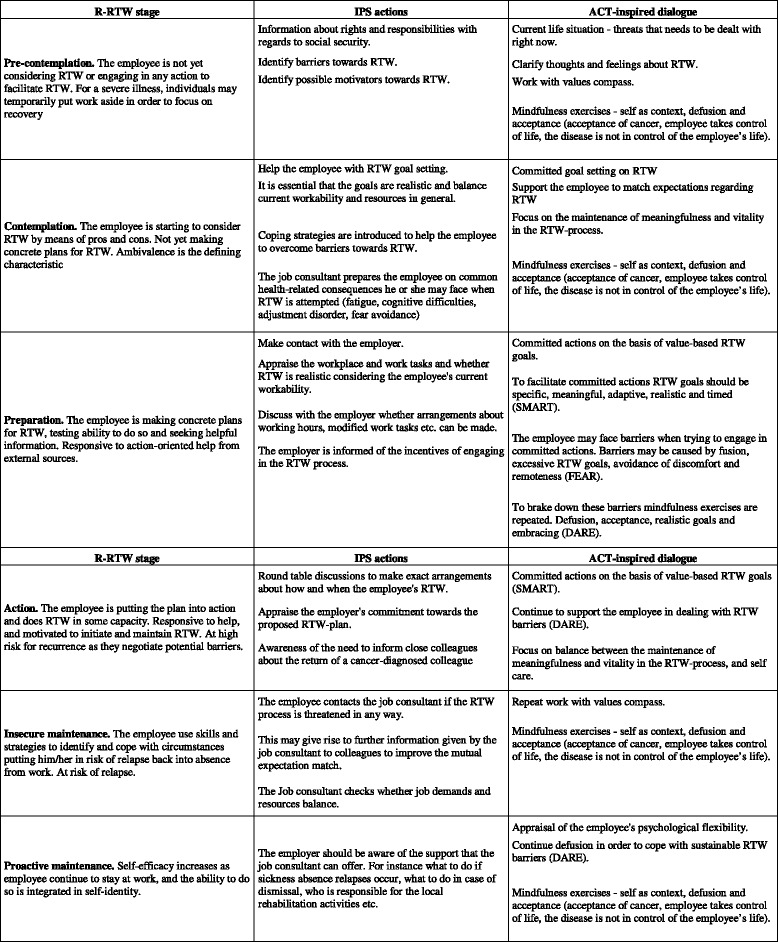
The structure used by the job consultants to tailor and support the return to work process. The individual rehabilitation plan is tailored according to different stages of readiness for return to work (R-RTW) and corresponding individual placement and support (IPS) actions that the job consultant initiates accompanied by acceptance and commitment therapy (ACT) inspired dialogue that should enhance commitment and change behaviour towards return to work (RTW)

At the first meeting with the job consultant the cancer survivor is asked to answer an online questionnaire. The questionnaire data is used to guide the job consultant regarding the cancer survivor’s readiness for RTW and need of support in order to set up an individual RTW plan. Hence, the ACT inspired dialogue and the systematic occupational needs assessment are integrated in the IPS phases by which the job consultant supports the survivor’s RTW-process (Fig. 1). The job consultant sets up meetings with the cancer survivor according to the RTW plan. Throughout the intervention the rehabilitation plan is adjusted to the survivors’ needs, actual resources and readiness for RTW. The initial rehabilitation plan and the subsequent actions and adjustments are registered online alongside the questionnaire data by the job consultants.

The intervention continues for a maximum of one year or until RTW. The survivor answers the Readiness for RTW scale after three months and at one-year follow up.

### Online questionnaire

#### Co-morbidity

Eighteen co-morbidity questions asking whether the respondent suffer from or has suffered from (asthma, allergies, diabetes, hypertension, coronary thrombosis, angina, cerebral thrombosis, chronic obstructive pulmonary disease, osteoarthritis, rheumatoid arthritis, osteoporosis, cancer, migraine, minor psychiatric disorder, major psychiatric disorder, herniated disc or other back disorders, cataract, tinnitus). There are four response categories (no/never, yes/present, yes/previously, if previously yes - do you have any late complications?). Number of co-morbidities are counted if present or previously present and give late complications [50, 51].

#### Work intention

One question about intention to work (i) in your estimation, what are the chances that you will be able to work in six months?” measured with a 10-point rating scale ranging from 1 (no chance) to 10 (very large chance) [52].

#### Self-efficacy

The seven-item general self-efficacy scale from The Copenhagen Psychosocial Questionnaire (COPSOQ) (i) I can always manage to solve difficult problems if I try hard enough, (ii) if someone opposes me, I can find the ways and means to get what I want, (iii) I am certain that I can accomplish my goals, (iv) thanks to my resourcefulness, I can handle unforeseen situations, (v) I can remain calm when facing difficulties because I can rely on my coping abilities, (vi) when I am confronted with a problem, I can find several solutions, (vii) I can handle whatever comes my way. The response categories are (not at all true, barely true, moderately true, and exactly true) which were scored from 0–100 with 100 representing the highest degree of self-efficacy [53].

#### Social support

From the 2nd version of COPSOQ, three items about social support from colleagues and supervisors, respectively; (i) how often do you get help and support from your colleagues?, (ii) how often are your colleagues willing to listen to your problems at work?, (iii) how often do your colleagues talk with you about how well you carry out your work? and (i) how often is your nearest superior willing to listen to your problems at work?, (ii) how often do you get help and support from your nearest superior?, (iii) how often does your nearest superior talk with you about how well you carry out your work?. The response categories are (always; often; sometimes; seldom; never/hardly ever; not relevant, which are scored from 0–100 with 100 representing the highest degree of support [54].

#### Readiness for RTW

Finally, the Readiness for RTW will be measured by the Danish version of the original R-RTW scale [40]. The instrument consists of two scales; 22 items for persons not yet returned to work: (i) you do not think you will ever be able to go back to work?, (ii) you have been making plans with someone from your workplace to return back to work?, (iii) you have been thinking about making some changes that will help you go back to work?, (iv) as far as you are concerned there is no point in thinking about returning to work?, (v) you have learned different ways to cope with your pain so you can return to work?, (vi) you are actively doing things now to get back to work?, (vii) you think you might be ready to go back to work?, (viii) you are planning to go back to work, even if your pain is not 100 % gone?, (ix) physically, you are starting to feel ready to go back to work?, (x) you have been increasing your activities at home in order to build up your strength to go back to work?, (xi) you are getting help from others to return to work?, (xii) you are not ready to go back to work?, (xiii) you have found strategies to make your work manageable so you can return to work?, (xiv) mentally, you are starting to feel ready to go back to work?, (xv) you have been wondering if there is something you could do to return to work?, (xvi) you worry about having to stop working again due to your injury?, (xvii) you have started thinking about going back to work”?, (xviii) you have a date for your first day back at work?, (xix) you wonder if you will be able to go back to work?, (xx) you wish you had more ideas about how to get back to work?, (xxi) you would like to have some advice about how to go back to work?, (xxii) as far as you are concerned, you do not need to go back to work ever”?.

And a scale with 12 items for those who have returned to work (part-time or full-time), but are at risk of sickness absence relapse: (i) you are trying different strategies to stay at work?, (ii) you are doing everything you can to stay at work?, (iii) you are getting help from others to stay at work?, (iv) you are working hard to find ways to cope with the difficulties of being back at work?, (v) you have learned different ways to cope with your pain so that you can stay at work?, (vi) you are taking steps to prevent having to go off job again due to your injury?, (vii) you have found strategies to make your work manageable so you can stay at work?, (viii) you are back at work but are not sure you can keep up the effort?, (ix) you worry about having to stop working again due to your injury?, (x) you still find yourself struggling to stay at work due to the effects of your injury?, (xi) you are back at work and it is going well?, (xii) you feel you may need help in order to stay at work?. Each item is scored on a five point ordinal scale (1 = strongly disagree) to (5 = strongly agree). Mean scores are calculated and forms four underlying stages for those not returned to work: Precontemplation, Contemplation, Preparation for action - self-evaluative, and Prepared for action - behavioural. And two stages for those already returned to work: Uncertain maintenance and proactive maintenance. The stage obtaining the highest mean score is interpreted as the current stage of readiness. The score of the R-RTW scale is immediately calculated in the online questionnaire. Measurements will be repeated after three and twelve months.

### Outcomes

#### Primary

The primary outcome is RTW among cancer survivors in the intervention group compared with the control group.

RTW is defined by at least 4 consecutive weeks of no social transfer payments or attending a modified job called “flexi job”.

The outcome is identified in a national register on weekly public transfer payments called the Danish Register for Evaluation of Marginalisation (DREAM) which has been found to provide valid information on labour market outcomes [55, 56]. Four consecutive weeks of no social transfer payments are considered to be equivalent to RTW along with four consecutive weeks of transfer payment equivalent to attending a “flexi job”.

#### Secondary

The secondary outcomes are whether socioeconomic status or co-morbidity is modifying the effect of the intervention on RTW.

Socioeconomic status is defined by type of work, educational level and last year household income before taxes, which are collected from Statistics Denmark [57].

Co-morbidity is defined by the Charlson-index; a weighted index including the number and seriousness of co-morbid diseases [58]. Data on diagnosis used in the Charlson-index are collected from the National Patient Registry in Denmark [59].

### Sample size calculation

The sample size calculation is based on the primary outcome RTW.

Approximately 450 patients are annually referred to the oncology ward with the eligible cancer diagnosis of which 20 % reside in the two intervention municipalities. It is expected that the employment rate among cancer patients would correspond to that of the Danish background population equivalent to approximately 80 % [60]. Furthermore, it was anticipated that 80 % gave informed consent to participate in the study. Hence, an expected number of 288 cancer patients per year. We estimated a mortality rate of 10 % during the one-year follow-up. Based on previous studies the RTW rate among cancer survivors who underwent occupational rehabilitation is approximately 82 % [18], and that the share will be approximately 60 % in the control group [61, 62]. The hazard ratio (HR) is then given by: (HR: ln (40 %) / ln (18 %) = 1.87). With a power of 90 % and a significance level of 5 % a required sample size of 290 patients is needed. To obtain sufficient strength in the adjusted analyzes 250 survivors should RTW. Thus, the total number of cancer survivors required is 430 (of which 90 reside in the two intervention municipalities) which means an inclusion period of 1.5 years.

### Statistical analyses

#### Primary outcome

The cumulative incidence proportion (CIP) as a function of the number of follow-up weeks is estimated using the Kaplan-Meier curve. The relative cumulative incidence of RTW within 52 weeks will be analyzed in a generalised linear regression model using the pseudo values method [63, 64].

Entry time is defined as the end of cancer treatment (identified from the hospital records) and the end of follow-up is 52 weeks following entry time. The outcome variable RTW consists of two measures: an event indicator (yes or no) and the time until RTW is identified in the DREAM register [65] or end of follow-up / competing risks (*early retirement benefit, retirement pension or death*)/censored observations (*emigration*), whichever comes first.

Adjustments for gender, age, cancer diagnosis, time since diagnosis and Charlson’s co-morbidity index [58] are going to be carried out. Moreover, analyses of whether socio-economic status or Charlson’s co-morbidity index modifies the effect of the intervention on RTW will also be carried out.

The significance level is set at p < 0.05. The results will be shown as crude and adjusted relative cumulative incidences, i.e. risks (RR) and corresponding 95 % confidence intervals (95 % CI).

STATA version 13.1 will be used as statistical software.

### Ethical considerations

The study has been notified to and registered by the Danish Data Protection Agency (nr. 1-16-02-657-14). Approval from the Danish National Committee on Biomedical Research Ethics was not relevant as this is only provided for projects using biological material. Furthermore, the present study does not include biomedical treatment.

All patients provided informed consent prior to inclusion in the study.

## Discussion

This controlled trial uses the existing setting by which occupational/vocational rehabilitation takes place in Danish municipalities as of today. Already job consultants are appointed the role as case managers in cases of long-term sickness absence. Sick leave due to cancer is however, dealt with in different ways compared to less serious conditions; partly because of taboo, reluctance of discussing work with a potentially fatally ill absentee and off cause due to compassion for this person. However, the social security system, i.e. the Sickness Benefit Act and whether the cancer survivor is entitled to receive sickness benefit may not differentiate between the reasons for sick leave. As a consequence the cancer survivor may lose financial security while being sick-listed and also lose her or his job in the long run [7, 26, 27]. Moreover, the cancer survivor is left on her or his own to deal with these challenges, despite the fact that the majority of cancer survivors are motivated for and wishes to RTW [11].

The innovative element of this intervention is the timing and early onset of the occupational rehabilitation which is much earlier than usual and that the job consultants are articulating work and take action on the cancer survivors’ R-RTW and support them in behaviour change towards RTW. The evaluation of this intervention will point out if this approach in deed increases the RTW chance compared to the control municipalities.

The intervention will also be evaluated through qualitative interviews in terms of its acceptability to stakeholders and whether the existing setting is appropriate and whether job consultants are capable of this task.

The knowledge of RTW facilitators and barriers are quite extensive [2]. However, in the present trial it was not possible for us to take into account all of these factors. The cancer-related symptoms and possible disabilities due to treatment are not addressed directly within our intervention protocol. As the intervention takes place parallel with cancer treatment all of the cancer survivors are in contact with the health care system. If the job consultants identify health-related barriers in relation to RTW, they are intended to suggest the cancer survivor to get in contact with the treating oncologist or the general practitioner (GP). As well as the job consultants themselves can contact the health care professionals for advice.

According to the integrated cancer pathway “Rehabilitation and Palliation in Cancer” [16]; each municipality in Denmark is obliged to provide cancer rehabilitation. The municipal job consultants are well informed about the local offered rehabilitation within their municipality. Therefore we anticipate that health-related issues will be addressed because the healthcare stakeholders are close to the survivor and to the job consultants.

The theory behind our hypothesis that the intervention will increase RTW chance originates from the stages of change model [44]. The R-RTW model addresses the motivational factors of behaviour change towards RTW and maintenance of work and was validated in a population of claimants with muscular-skeletal disorders [40]. The scale has been translated to Norwegian and was validated in a Norwegian inpatient occupational rehabilitation program in sick-listed people [66]. The scale was reliable and a valid tool for the RTW prognosis.

In Denmark the R-RTW scale is currently being validated. The data from this study will be used in the validation of responsiveness. It was not possible to obtain R-RTW scores from the control group in this study. Thus, the scores obtained from the cancer survivors in the intervention group are used solely by the two job consultants to adjust their rehabilitation to the right stage.

### Methodological considerations

The golden standard when effects of an intervention are to be assessed is the randomised controlled trial (RCT) [18]. However, when studies are conducted in real settings, like in municipal job centres; recently published articles from the Danish national return-to-work program showed challenges in applying a randomised study design; i.e. spill over effects between study arms within the municipality [67–69]. Therefore we chose a controlled study design. When the control municipalities are to be chosen, high priority will be given to municipalities that resemble the RTW rate of the intervention municipalities the year before our study was initiated, i.e. 2013. For that purpose official rates from Statistics Denmark [57] will be used. We may not be able to identify municipalities which also can supply sufficient numbers of cancer patients fulfilling the inclusion and exclusion criteria. We might need to compromise on which municipalities to include as controls to get sufficient numbers of cancer survivors, which in turn might give residual confounding that will lower the internal validity of our results.

We believe that if the job consultants are to succeed in their support and help to the cancer survivors and thereby increase the R-RTW; they must identify barriers and facilitators in the RTW process and use the ACT inspired dialogue. Otherwise they may not be able to engage the cancer survivors in committed actions that are value-based and make sense to the survivors’ perception of quality of life (including work). In order for us to keep track of the compliance with the study protocol; the job consultants attend monthly supervisions with the psychologist who trained them in the use of ACT and IPS. Because the intervention takes place in the job consultants usual work setting, we fear that they are at risk of resuming old work habits and thereby violate the innovative elements in our intervention. We anticipate that the supervisions act as an arena where experiences are shared and difficult situations are discussed in order for the job consultants to learn and cope with their new job tasks and demands.

Due to the described intervention given by the job consultants it is not possible to blind the cancer survivors nor the job consultants or the person who will perform the analyses.

The sample size calculations are based on cancer survivors being sick-listed at entry time in the analyses. This means that we may need more than 430 cancer survivors in total. This might lead to a longer inclusion period than the anticipated 1.5 years. We anticipate that results will be available ultimo 2017.

## Abbreviations

ACT: Acceptance and commitment therapy
CI: Confidence interval
CIP: Cumulated incidence proportion
COPSOQ: Copenhagen psychosocial questionnaire
DREAM: Danish register for evaluation of marginalisation
GP: General practitioner
HR: Hazard ratio
IPS: Individual placement and support
RCT: Randomised controlled trial
RR: Relative risk
R-RTW: Readiness for return to work
RTW: Return to work
